# Supplementary material for: A retrospective study of consistency between immunohistochemistry and polymerase chain reaction of microsatellite instability in endometrial cancer
Source: PeerJ. 2023 Aug 28;11:e15920. doi: 10.7717/peerj.15920 (PMC10470453; doi:10.7717/peerj.15920)
Supplement: Supplemental Information 1 — MSP, methylation specific PCR; UMSP, unmethylation specific PCR [file peerj-11-15920-s001.docx]

**Supplemental Table 1**. Sequences of primers

| **PCR** | **Primer name** | **Sequence (5'-3')** |
| --- | --- | --- |
| MSP in promoter region |  |  |
|  | MSP-F | ACGTAGACGTTTTATTAGGGTCGC |
|  | MSP-R | CCTCATCGTAACTACCCGCG |
| UMSP in region |  |  |
|  | UMSP-F | TTTTGATGTAGATGTTTTATTAGGGTTGT |
|  | UMSP-R | ACCACCTCATCATAACTACCCACA |

Notes.

MSP, methylation specific PCR; UMSP, unmethylation specific PCR
