# Supplementary material for: A retrospective study of consistency between immunohistochemistry and polymerase chain reaction of microsatellite instability in endometrial cancer
Source: PeerJ. 2023 Aug 28;11:e15920. doi: 10.7717/peerj.15920 (PMC10470453; doi:10.7717/peerj.15920)
Supplement: Supplemental Information 2 — EC, endometrioid carcinoma; SC, serous carcinoma; CC, clear cell carcinoma; UC, undifferentiated carcinoma of the endometrium; CS, carcinosarcoma; MC, mixed carcinoma; MLA, mesonephric-like adenocarcinomas; FIGI, International Federation of Gynecology and Obstetrics; LVSI, lymphovascular invasion; MMR, mismatch repair; dMMR, mismatch repair deficient; pMMR, mismatch repair proficient; MSI, microsatellite instability; MSS, microsatellite stable; MSI-L, microsatellite instability-low; MSI-H, microsatellite instability-high. [file peerj-11-15920-s002.docx]

**Supplemental Table 2**. Patient demographic information and clinicopathologic features (n=333).

|  | **Overall** |
| --- | --- |
|  | **(n=333)** |
| **Age** |  |
| Mean (SD) | 53.7 (9.84) |
| Median [Min, Max] | 53.0 [25.0, 87.0] |
| **Specimen** |  |
| Curettage | 131 (39.3) |
| Hysterectomy | 202 (60.7) |
| **Family history** |  |
| No | 224 (67.3) |
| Yes | 30 (9.0) |
| Unknown | 79 (23.7) |
| **FIGO** |  |
| I | 249 (74.8) |
| II | 22 (6.6) |
| III | 51 (15.3) |
| IV | 11 (3.3) |
| **Histology** |  |
| EC | 279 (83.8) |
| MC | 23 (6.9) |
| SC | 15 (4.5) |
| CS | 6 (1.8) |
| CC | 5 (1.5) |
| UC | 4 (1.2) |
| MLA | 1(0.3) |
| **Grade** |  |
| G1 | 176 (52.9) |
| G2 | 65 (19.5) |
| G3 | 92 (27.6) |
| **LVSI** |  |
| No | 247 (74.2) |
| Yes | 86 (25.8) |
| **Muscular invasion** |  |
| Superficial | 244 (73.3) |
| Deep | 89 (26.7) |
| **Lymphatic metastasis** |  |
| No | 297 (89.2) |
| Yes | 36 (10.8) |
| **MMR status** |  |
| dMMR | 84 (25.2) |
| pMMR | 249 (74.8) |
| **MSI status** |  |
| MSS | 249 (74.8) |
| MSI-L | 4 (1.2) |
| MSI-H | 80 (24.0) |

EC: endometrioid carcinoma; SC: serous carcinoma; CC: clear cell carcinoma; UC: undifferentiated carcinoma of the endometrium; CS: carcinosarcoma; MC: mixed carcinoma; MLA: mesonephric-like adenocarcinomas, FIGI: International Federation of Gynecology and Obstetrics, LVSI: lymphovascular invasion, MMR: mismatch repair, dMMR: mismatch repair deficient, pMMR: mismatch repair proficient, MSI: microsatellite instability, MSS: microsatellite stable, MSI-L: microsatellite instability-low, MSI-H: microsatellite instability-high.
