# Supplementary material for: A retrospective study of consistency between immunohistochemistry and polymerase chain reaction of microsatellite instability in endometrial cancer
Source: PeerJ. 2023 Aug 28;11:e15920. doi: 10.7717/peerj.15920 (PMC10470453; doi:10.7717/peerj.15920)
Supplement: Supplemental Information 3 — MMR, mismatch repair. [file peerj-11-15920-s003.docx]

**Supplemental Table 3**. The results of four MMR protein expressions detected in curettage and hysterectomy specimens.

|  | Curettage specimens (n=131) | | Hysterectomy specimens (n=202) | |
| --- | --- | --- | --- | --- |
|  | Retain (%) | Lost (%) | Retain (%) | Lost (%) |
| MLH1 | 116(84.7) | 21(15.3) | 173(85.6) | 29(14.4) |
| PMS2 | 112(81.8) | 25(18.2) | 170(84.2) | 32(15.8) |
| MSH6 | 126(92.0) | 11(8.0) | 186(92.1) | 16(7.9) |
| MSH2 | 129(94.2) | 8(5.8) | 191(94.6) | 11(5.4) |

Notes.

MMR: mismatch repair.
