# Supplementary material for: A retrospective study of consistency between immunohistochemistry and polymerase chain reaction of microsatellite instability in endometrial cancer
Source: PeerJ. 2023 Aug 28;11:e15920. doi: 10.7717/peerj.15920 (PMC10470453; doi:10.7717/peerj.15920)
Supplement: Supplemental Information 4 — MMR, mismatch repair; dMMR, mismatch repair deficient; pMMR, mismatch repair proficient; MSI, microsatellite instability; MSI-H, microsatellite instability-high; MSS/MSI-L, microsatellite stable/microsatellite instability-low. [file peerj-11-15920-s004.docx]

**Supplemental Table 4**. The MMR or MSI status of curettage and hysterectomy specimens.

|  | MMR | | MSI | |
| --- | --- | --- | --- | --- |
|  | dMMR (%) | pMMR (%) | MSI-H (%) | MSS/MSI-L (%) |
| Curettage specimens (n=131) | 36 (27.5) | 95 (72.5) | 35 (26.7) | 96 (73.3) |
| Hysteretomy specimens (n=202) | 48 (23.8) | 154 (76.2) | 45 (22.3) | 157 (77.7) |

Notes.

MMR: mismatch repair, dMMR: mismatch repair deficient, pMMR: mismatch repair proficient, MSI: microsatellite instability, MSI-H: microsatellite instability-high, MSS/MSI-L: microsatellite stable/microsatellite instability-low.
